# Predicting sensory evaluation of spinach freshness using machine learning model and digital images

**DOI:** 10.1371/journal.pone.0248769

**Published:** 2021-03-19

**Authors:** Kento Koyama, Marin Tanaka, Byeong-Hyo Cho, Yusaku Yoshikawa, Shige Koseki

**Affiliations:** Graduate School of Agricultural Science, Hokkaido University, Sapporo, Japan; Ton Duc Thang University, VIET NAM

## Abstract

The visual perception of freshness is an important factor considered by consumers in the purchase of fruits and vegetables. However, panel testing when evaluating food products is time consuming and expensive. Herein, the ability of an image processing-based, nondestructive technique to classify spinach freshness was evaluated. Images of spinach leaves were taken using a smartphone camera after different storage periods. Twelve sensory panels ranked spinach freshness into one of four levels using these images. The rounded value of the average from all twelve panel evaluations was set as the true label. The spinach image was removed from the background, and then converted into a gray scale and CIE-Lab color space (L*a*b*) and Hue, Saturation and Value (HSV). The mean value, minimum value, and standard deviation of each component of color in spinach leaf were extracted as color features. Local features were extracted using the bag-of-words of key points from Oriented FAST (Features from Accelerated Segment Test) and Rotated BRIEF (Binary Robust Independent Elementary Features). The feature combinations selected from the spinach images were used to train machine learning models to recognize freshness levels. Correlation analysis between the extracted features and the sensory evaluation score showed a positive correlation (0.5 < *r* < 0.6) for four color features, and a negative correlation (‒0.6 < *r* < ‒0.5) for six clusters in the local features. The support vector machine classifier and artificial neural network algorithm successfully classified spinach samples with overall accuracy 70% in four-class, 77% in three-class and 84% in two-class, which was similar to that of the individual panel evaluations. Our findings indicate that a model using support vector machine classifiers and artificial neural networks has the potential to replace freshness evaluations currently performed by non-trained panels.

## Introduction

Freshness of food heavily influences consumer food selection [1–3]. However, defining freshness is difficult, as its assessment depends on individual experience [4,5] and the type of food being evaluated [6]. Chemical components, such as propanethial S-oxide in scallions [7], asparagine, the amino acid content of asparagus [8], and the chlorophyll fluorescence of harvested broccoli [9], are considered as indicators of freshness. However, the concept of freshness as defined in previous studies is not practical for its evaluation due to these chemical components being chosen arbitrarily as freshness indicators without taking subjective consumer perceptions into account.

Studies on consumer perception of produce freshness have focused on broader concepts of freshness [10]. Various studies have attempted to combine consumer evaluations with objective indices [6,10–12]. In these studies, measurable standards, such as changes in the color and weight of food, play an important role in interpreting the results of sensory evaluation tests conducted by a panel. For example, Jung (2012) reported that the rate of weight loss of spinach was strongly correlated with its freshness, as evaluated by a panel. In contrast, this subjective freshness cannot be directly predicted by physicochemical properties, since physicochemical properties do not directly reflect consumer perception.

As sensory evaluation is time-consuming and expensive, predicting freshness using machine-based strategies would be more efficient for food categorization than panel-based sensory evaluation. Once a model learned sensory evaluation score, the model would reproduce classification of freshness of food from the same concept of human sensory evaluation many times without further panel labor. Machine learning has been previously used to predict the freshness of fish [13,14] and puffed snacks [15], as well as evaluate the quality of olive oil [16], using the learning results of sensory evaluation. Image processing (visual), microphone sensors (sound), and texture profile analysis (touch) for the quantification of human perception were applied to develop these prediction models. Visual perception is important for humans when evaluating product freshness [11,17]. Recently, digital image in smartphone camera has been used for classification of freshness of fish and squid by machine learning [18,19]. Image analysis and machine learning have also demonstrated the ability to predict food quality and freshness using visual perception.

A wide variety of methods such as K-Nearest Neighbor, support vector machine, Artificial Neural Network, Convolutional Neural Network have been used for classification in food quality evaluation in computer vision system [20]. The support vector machine (SVM) model is a machine learning method that can be used for both regression and classification. The SVM classifier has been applied to various fields, such as the detection and classification of plant diseases [21], and the evaluation of tomato ripeness [22]. An artificial neural network (ANN) is a computational modeling tool that has been widely accepted in a range of situations modeling complex real-world problems [23]. The SVM model and ANN are flexible and are endowed with a nonlinear learning capability [24,25], as observed in human perceptions [26]. As a result, machine learning methods have been applied to the field of fresh product classification in recent years [27].

Spinach, wild rocket, and baby leaf are popular leafy vegetables that tend to wilt during storage. Thus, freshness is an important factor for leaf vegetables. Machine learning with a machine vision system can predict the freshness of spinach and fresh-cut iceberg lettuce by learning sensory evaluation results [28,29]. Although trained panels are widely used for the sensory evaluation from which a machine learning model is developed, the perception of trained panels is not representative of the perception of general consumers when measuring preferences and the acceptance of a product [30,31]. Thus, using non-trained panels to assess freshness is important to help improve our understanding of freshness from a consumer perspective.

Spinach was used to assess freshness by machine learning due to its popularity, quick degradation compared to other vegetables, and the visibility of degradation (i.e. wilted leaves), which can be detected by image recognition. The objective of this study was to evaluate spinach freshness using an SVM classifier and an ANN. Spinach leaf images were taken, and a sensory evaluation was conducted by non-trained panels to obtain data for the machine-based evaluation model. Predicting the results of the visual evaluation of freshness by humans using machine learning methods and smartphone images is cost effective, rapid, and reproducible at any time or place.

## Materials and methods

### Spinach sampling

A total of 100 spinach (*Spinacia oleracea*) plants cultivated in Hokkaido in Japan were bought from a supermarket (AEON) in Hokkaido and used as samples in this study. The heads of spinach were divided into individual plastic bags (273 × 268 × 0.06 mm) and stored at 10°C for 12 days.

### Image acquisition system

The image acquisition system consisted of two components: a smartphone camera, an illumination chamber with a lighting system. A cardboard box (336×434×200 mm) was used as the illumination chamber. Two LED lights were placed on the left and right sides of the box ceiling and used to irradiate objects during image acquisition to maintain reflectance. The smartphone (iPhone 5c, Apple, Cupertino, USA) used to take spinach images was fixed at a vertical distance of 200 mm above the bottom of the cardboard box. One image was taken for each spinach sample using the camera specifications summarized in Table 1. Images of one randomly selected leaf from each spinach sample were taken every day to obtain a total of 1,045 images for 12 days. The acquired images were transferred to a computer for sensory evaluation and feature extraction. These images were used for sensory evaluation, as previously reported [32].

**Table 1:** Camera settings used to obtain spinach images.

Variable	Specification
Image size	3264 × 2448 pixels
Zoom	No zoom
Flash mode	No flash
Sensitivity	Auto
White balance	Auto
Operation mode	Auto
Aperture	f/2.4
Exposure time	Auto
Image type	JPEG
Focal length	4.12 mm
Resolution	72 dpi

### Sensory evaluation

#### Development of sensory evaluation guidelines

**Fig 1:**
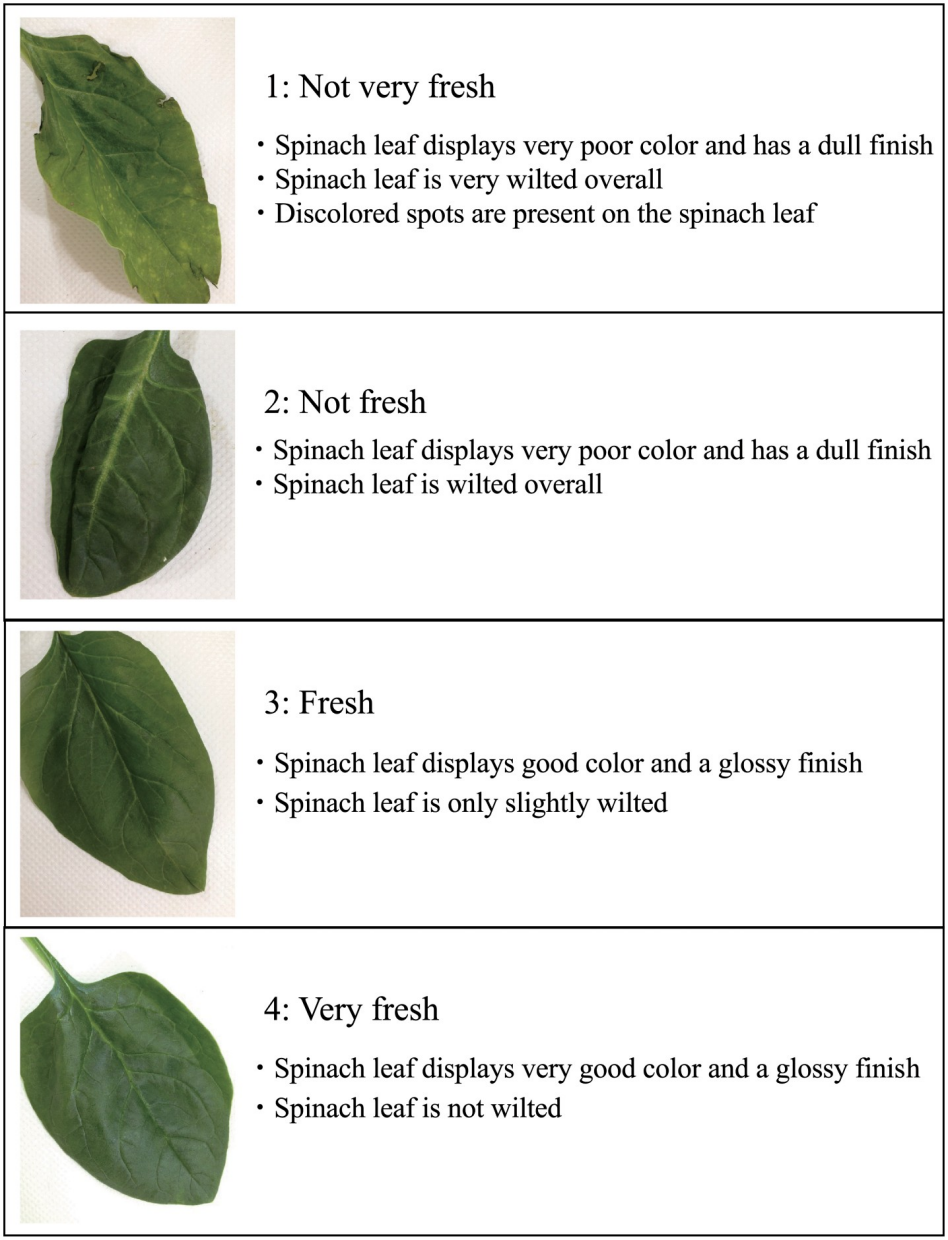
Spinach freshness level guidelines for sensory evaluation.

A guideline for the sensory evaluation of spinach freshness was developed to reduce errors between subjects, as shown in Fig 1. Freshness was classified into one of four levels (1, not very fresh; 2, not fresh; 3, fresh; and 4, very fresh), as panels can effectively classify the freshness of fruits and vegetables into a maximum of five levels [12]. In addition, we conducted a survey of consumer visual perception of spinach freshness to determine the reference images for the guideline. Thirty spinach images were classified by 16 subjects (6 females and 10 males; age range, 21–46 years; mean age, 24.8 years) into the four freshness levels. All the subjects were non-trained panel and evaluated subjectively freshness of spinach. The reference images were determined based on the survey results.

#### Evaluation procedure

Twelve subjects participated in the sensory evaluation (4 females and 8 males; age range, 21–28 years; mean age, 23.3 years). None of the subjects were experts in sensory evaluation. Before sensory evaluation, the subjects were introduced to the evaluation procedure and provided with the reference guidelines (Fig 1). Individuals sample images were shown to the subjects on a display. Reference images were presented at the same time as the sample. The subjects performed the evaluation by referring to the guidelines using a four-level scale. The order effect was eliminated as described by Arce-Lopera et al. (2013). A total of 100 to 145 samples were used for each sensory evaluation, provided in a random order for each subject. The subjects performed the sensory evaluation 10 times, evaluating 100 to 145 samples each time, until 1,045 samples were evaluated in total. All the subjects evaluated the same 1,045 images of spinach samples. The mean sensory evaluation value of the 12 subjects was used as the true label. Here, *y*_*i*,*j*_ is a sensory evaluation score for image *i* by subject *j*. True label of image *i* Y_*i*_ is calculated as follows:

$$Y_{i}=\frac{1}{n_{j}}\sum_{j=1}^{n_{j}}y_{i,j}$$

where *n*_*j*_ is a total number of subjects.

### Image processing and feature extraction

#### Background removal and color extraction

**Fig 2:**
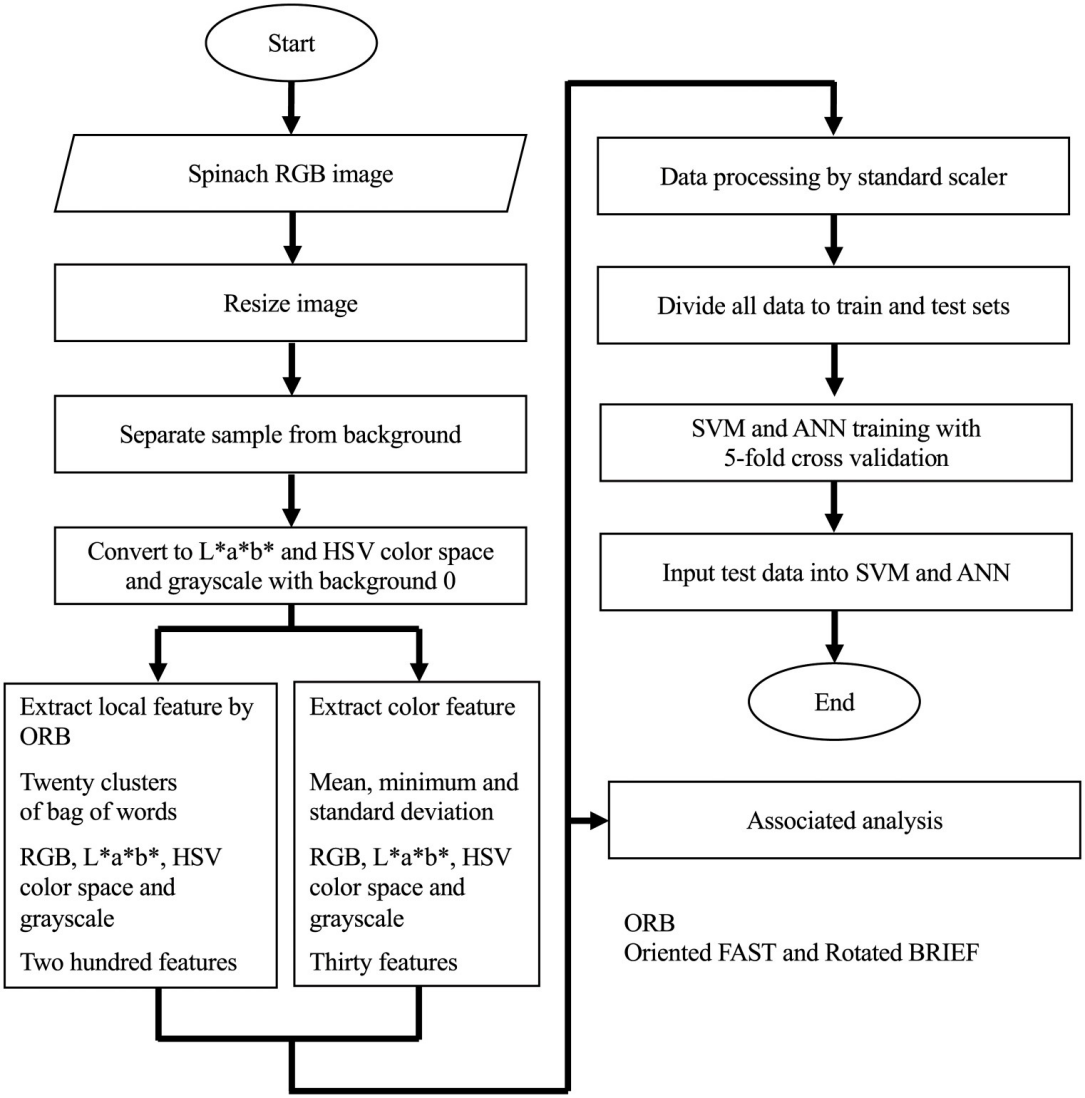
Flow chart of the algorithm used to evaluate the color and local features of spinach samples extracted from smartphone images.

Fig 2 shows the image pre-processing steps used to extract color and local features calculated by the k-mean clustering of the Oriented FAST and rotated BRIEF [33] features. In the first step, images were resized to 490 × 653 pixels, and the colored parts of the images were converted from RGB to grayscale. Then, the spinach leaves were separated from the background. The optimal threshold of gray-scale intensities was calculated based on binary inverting and Otsu algorithms [34] and applied to the grayscale image to separate the spinach leaves from the background. The grayscale image was assumed to consist of two classes of histograms, and the optimal threshold that minimizes the intra-class variance was searched in the Otsu algorithms. To extract color information from the samples and remove background-related data, the binary image and the original RGB image were superimposed via logical and operator actions. As a result, the spinach leaves and background had RGB images with color values and zeros, respectively. The images were converted from RGB into grayscale, CIE-Lab color space (L*a*b*), and Hue, Saturation and Value (HSV), which are often used in food computer vision [35,36]. The mean value, minimum value, and standard deviation of each component of color were calculated from the spinach image without background. Three color features are extracted for each component of the color. Thus, thirty color features were extracted for ten components of color.

#### Extraction of key points from an image by ORB

The local features for assessing the similarity of images are key points. The key points are small patches of an image that differ from the surrounding pixels. Scale invariant feature transform (SIFT) [37,38] is a widely used algorithm to determine key points in computer vision. However, ORB (Oriented FAST and Rotated BRIEF) [33] was chosen as our algorithm to extract key points in images since, contrary to SIFT, ORB is free from licensing restrictions and has a fast computation. Moreover, ORB has been used for detecting the local damaged strawberry area [39]. ORB was also used to detect the local texture of the spinach leaves.

The ORB algorithm is a mixture of modified FAST (Features from Accelerated Segment Test) [40] detection and direction-normalized BRIEF (Binary Robust Independent Elementary Features) [41] description. FAST corners are detected in each layer of the scale pyramid for multi-scale robustness. Cornerness was evaluated using the Harris Corner measure (A Combined Corner and Edge Detector) [42] to order the FAST key points. Because FAST does not produce orientations, the intensity centroid technique was used [33]. The intensity centroid constructs a vector from the corner’s center to a centroid, which is then used to compute the orientation. BRIEF [41] is a feature point descriptor that takes all of the key points found by the FAST algorithm and converts it into a binary feature vector. A steered BRIEF descriptor was employed for stability with rotation. ORB features are invariant to scale and rotation. In the present study, the ten ORB detectors were developed at grayscale and each RGB, L*a*b*, and HSV color space, respectively.

#### Bag-of-words representation

We employed the bag-of-words approach [43] to describe local features from the image key points. Any image can be represented as a bag of visual words, which is more compact than simply representing the image via key points. A comparison of the visual words representing two images could be used to determine whether the two images are similar. Representing an image by a bag of visual words is performed as follows: First, the points of interest in the images from the training dataset are detected, and then the local description is computed by ORB. These local descriptions in key points are all grouped into a pre-defined number of clusters using the k-means clustering algorithm. The resulting cluster centers are treated as a dictionary of visual words. Finally, for each image, we created a frequency histogram from the vocabularies in the image. These histograms are our bag of visual words. Twenty clusters constructing a histogram are chosen for each component of color after a trial of different numbers of clusters. The ten dictionaries of visual words were developed at grayscale, and each RGB, L*a*b*, and HSV color space, using a training data set. Then, the local features for the training and testing sets were classified depending on the distance between the training or testing feature and the cluster center. Twenty categories in each component of color were used as local features. In total, 200 categories were used as local features because ten components of color were used. The script of the above color and local feature extraction is provided in the Supplementary Material.

### Machine learning model

Supervised learning was used to classify spinach freshness. The rounded value of the average from all 12 panel evaluations was set as the true label, while the features calculated from the computer vision system were used as input for the machine learning model. The global features were consistent with the sample color and local features. Three color features and twenty clusters in the bag of words as local features were extracted from each color component. All feature values, thirty color features, and two hundred local features were standardized by removing the mean and scaling to unit variance for every color feature and every bar in the histogram of local features. These were then used as the input for the machine learning model. A total of 230 input features were used to classify spinach freshness as representative stimuli from the computer vision system. SVM classification and ANN machine learning models were tested to develop the freshness prediction model. The SVM classification and ANN models were selected for developing the prediction model because the algorithms have the capability to classify targets with non-linear relationships [44]. Both models are widely used for learning tasks. Python 3.7 was used for data analysis. Both models were implemented using the Scikit-learn machine learning library.

#### Support vector machine (SVM) classifier

Support vector machines are supervised learning techniques used for developing classification and regression models. Multi-class SVM can be considered as an aggregation of multiple binary SVMs. SVM finds an optimal boundary between the labeled samples with the least error and maximum margin. The kernel function transforms training data into a space where the training data can be separated by a hyperplane. A nonlinear boundary is found by exploiting the kernel. Different kernel functions, including linear, polynomial, radial basis function, and sigmoid, were tested to verify the robustness of the classifier model. In the present study, a radial basis function (rbf) kernel was used to investigate the nonlinear relationships between inputs and outputs. The SVM hyperparameters in this study were selected using a grid search method and 5-fold cross validation. The parameter C, which determines the regularization strength, was 9, while the parameter gamma was 0.001. The script of the SVM classifier is provided in the Supplementary Material.

#### Artificial neural network model

The number of hidden layers and the number of neurons depends on the complexity of the learning task and the amount of training data. The number of nodes in the input layer was fixed at 230, the number of extracted features from the sample image. The number of nodes in the output layer was 4, which corresponds to the number of freshness levels. The ANN performance was tested according to the number of hidden layers. Based on the final results, four hidden layers were sufficient for this study. Four hidden layers were used, with the number of hidden units in each hidden layer being 32, 128, 128, and 64. Neural nets used hyperbolic tangent or sigmoid, but the Rectified Linear Unit typically learns much faster in artificial neural network. Thus, we used RelU as activation function in this study. The activation function were “sigmoid” in the first layer and “Rectified Linear Unit (ReLU)” in the second, third and fourth layers and “softmax” in the last layer [45,46]. Parameters including learning rate, epochs, and batch size on overall ANN performance were selected using a grid search method and 5-fold cross validation. In this study, Adam optimizer [47] was used, and the learning rate, dropout rate, epoch value, and batch size were set to 0.001, 0.1, 80, and 90, respectively. The script of the ANN model is provide in the Supplementary Material.

#### Model development and evaluation of model accuracy

The entire dataset (1,045 images) was randomly divided into the training set (80%) and testing set (20%). This split ratio (training:testing = 80:20) is commonly used in machine learning applications [44,48]. The number of images for train set and test set is 836 and 209, respectively. Since the number of labels was unbalanced between each label, stratified methods were used for sample separation. Then, 5-fold cross validation was used to set training data parameters. The test set was classified by the SVM classifier and ANN models, and the model accuracy for each class was calculated. The predicted 4-level class was then assigned to 3-level (1, not very fresh; 2, not fresh; and 3 and 4 fresh) and 2-level (1 and 2; not fresh; and 3 and 4 fresh) classes for further data analysis. All data processing was performed using Python 3.7.

### Statistical analysis

Statistical and correlation analyses for sensory evaluation were conducted using one-way ANOVA and bivariate analysis, respectively. The SPSS statistical package (IBM SPSS Statistics 20.0, IBM, USA) was used to analyze significance in this study. Standard deviations were calculated to examine the variability of the results of the evaluation by all subjects. Standard deviation of subjective evaluation $\overset{-}{\sigma }$ is described as follows:

$$\sigma_{i}=\sqrt{\frac{1}{n_{j}}\{{\left(y_{i,1}-\overset{-}{y}\right)}^{2}+{\left(y_{i,2}-\overset{-}{y}\right)}^{2}+\cdots +{\left(y_{i,n_{j}}-\overset{-}{y}\right)}^{2}\}}$$

$$\overset{-}{\sigma }=\frac{1}{n_{i}}\Sigma \sigma_{i}$$

where *n*_*i*_ is a total number of images. A one-way ANOVA with random blocks was used to evaluate the effect of error between subjects; it was analyzed using the same method as a repeated measures design with one repeated measures factor. Panel and spinach samples were used as factors in this study. In addition, the effect size (Cohen’s *f*^2^) of both the panel and sample was calculated. Effect sizes were defined at the following levels: small effect, 0.02; medium effect, 0.15; large effect, 0.35 [49]. Furthermore, a correlation analysis was used to determine whether the color and local features extracted from the sample images were related to the sensory evaluation results. Correlation coefficient *r* was calculated as follows:

$$r=\frac{\Sigma (x_{i}-\overset{-}{x})/(Y_{i}-\overset{-}{Y})}{\sqrt{{\Sigma (x_{i}-\overset{-}{x})}^{2}}∙\sqrt{{\Sigma (Y_{i}-\overset{-}{Y})}^{2}}}$$

where *x*_*i*_ is the color or local features extracted from image *i*.

**Fig 3:**
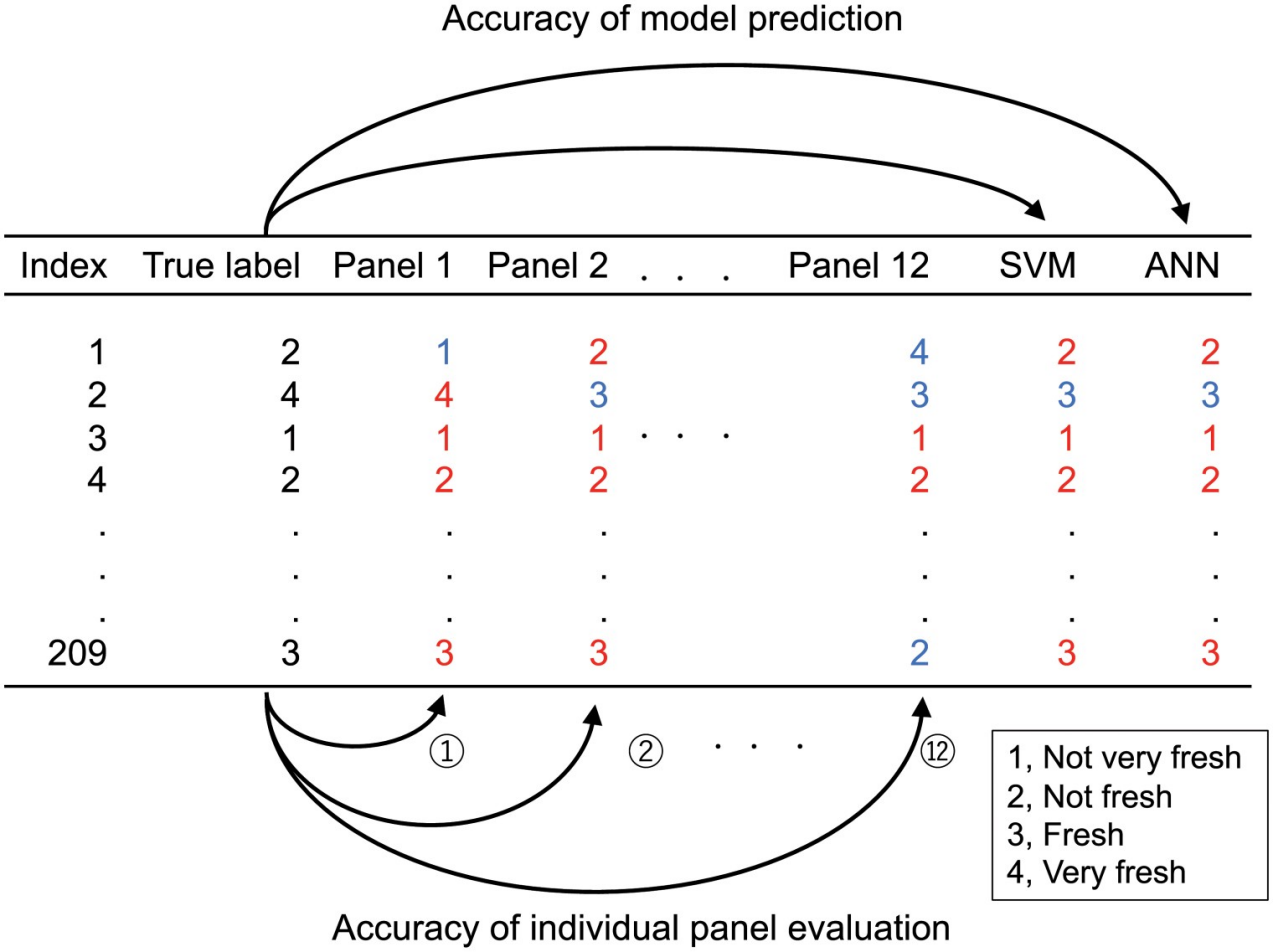
Accuracy of model and panels. Twelve accuracies were calculated for the individual panels. The red and blue numbers mean correct and incorrect answers. The true label is the rounded value of the average from all panel evaluations.

Individual panel evaluation was compared with the true label, the rounded value of the average from all panel evaluations (Fig 3). The test set was classified by the panels, and the accuracy of the individual panels for each class was calculated.

$$Accuracy\;(\%)=\frac{Number\;of\;correctly\;classified\;evaluations\;by\;panel\;j}{Number\;of\;evaluations}\times 100$$

In total, twelve panel accuracies were calculated. Accuracy of model prediction was also calculated as follows:

$$Accuracy\;(\%)=\frac{Number\;of\;correctly\;classified\;evaluations\;by\;a\;model}{Number\;of\;evaluations}\times 100$$

The evaluated 4-level class by panels was then assigned to 3-level (1, not very fresh; 2, not fresh; and 3 and 4 fresh) and 2-level (1 and 2; not fresh; and 3 and 4 fresh) classes for further data analysis. Lastly, we compared the accuracy between the model and panels to determine whether the model accuracy was equivalent to panel evaluation.

#### Ethical approval

Ethics committee in Research Faculty of Agriculture in Hokkaido University waived approval, because evaluation of spinach freshness had negligible risk and individual panels were non-identifiable through the obtained data. All the participants were orally informed that the results of evaluation of spinach freshness would be open in a research paper and the data would be analyzed anonymously. All the participants gave consent orally.

## Results

Of the 1,045 samples evaluated by 12 subjects into the four freshness levels, 172, 373, 452, and 48 samples were classified into freshness levels 1 (not very fresh), 2 (not fresh), 3 (fresh), and 4 (very fresh), respectively. Levels 2 and 3 included the largest number of samples (35.7% and 43.3%, respectively). The standard deviation between subjects was 0.61, indicating that the evaluation result variability of subject-based sensory evaluation was small compared to the size of the evaluation level. ANOVA showed a significant difference in freshness score due to both panel and sample differences (p < 0.01). The effect size was 0.13 for the panel and 1.77 for the sample, indicating that the effect of the difference between subjects on the evaluation was sufficiently small. Therefore, changes in the evaluation result were more affected by differences in the samples than in the panels. Correlation analysis between the color and local features obtained from the spinach image and the sensory evaluation score showed a positive correlation (0.5 < *r* < 0.6) for a minimum value of gray, g, v, and L*, and a negative correlation (‒0.6 < *r* < ‒0.5) for six clusters in the ORB local features. Freshness, as evaluated by the panel, was related to the color information and local features extracted from the spinach sample image.

**Fig 4:**
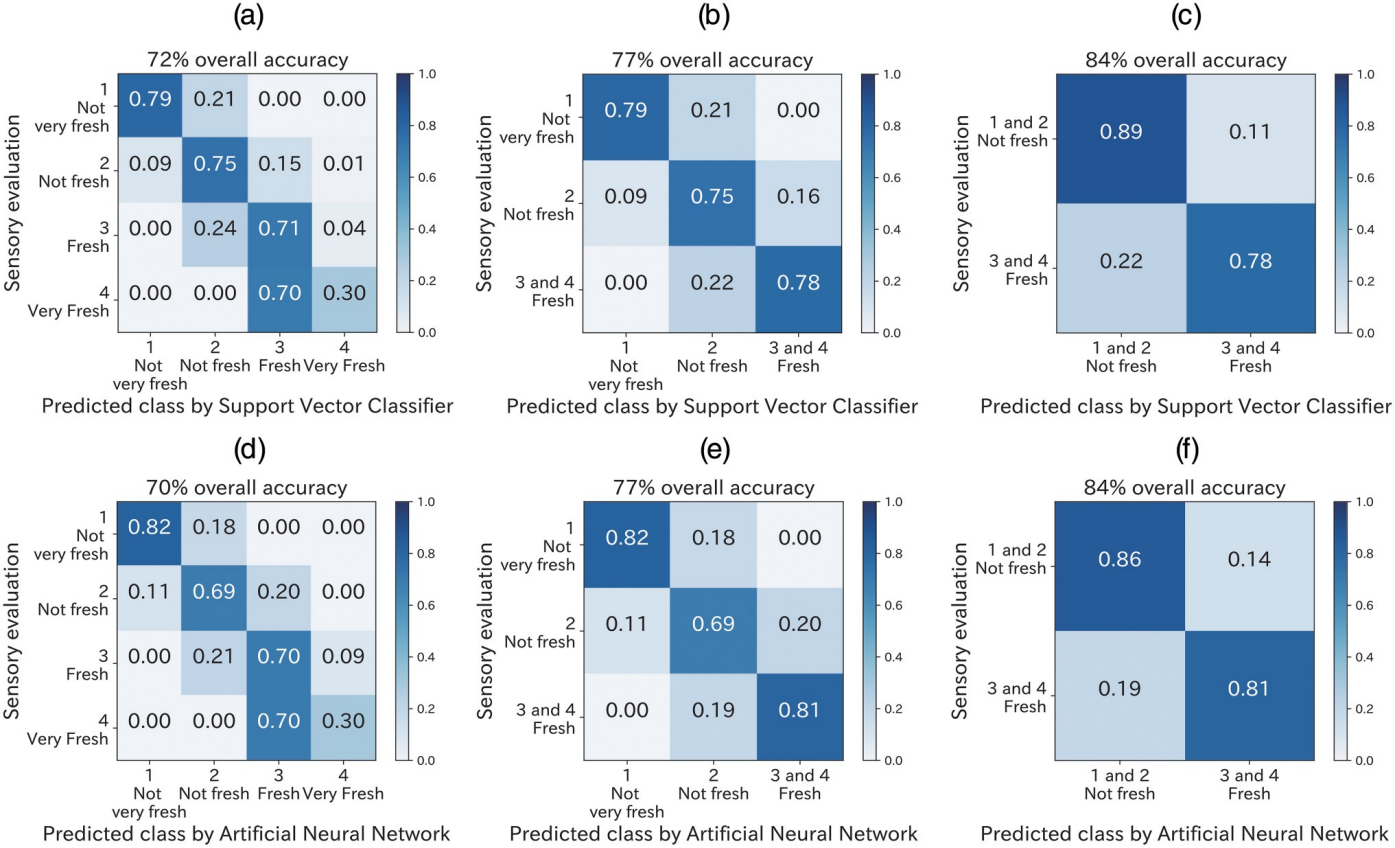
Test set confusion matrices of 5-fold cross validated SVM classifier and ANN models. True label is the mean value of the sensory evaluation: (a) a four-level classification, (b) a three-level classification, and (c) a two-level classification by SVM; (d) a four-level classification, (e) a three-level classification, and (f) a two-level classification by ANN.

The SVM classifier and ANN prediction results are shown in Fig 4, which contains a confusion matrix showing the percentage of true and false test sample predictions per level. The true label is the rounded value of the average from all panel evaluations. The lowest overall accuracy was 70%, using a four-level classification. We tried also 10-fold cross validation and obtained almost the same overall accuracy. The overall accuracy is only 1% difference between 5-fold cross validation and 10-fold cross validation results. The lower the class number, the higher the overall accuracy obtained. In the case of binary classification, the overall accuracy was 84% for both the SVM classifier and ANN model. Only one prediction were obtained with two level differences between the true label and model prediction (Fig 4). Only one image in 75 images (0.01%) of “2, not fresh” spinach was evaluated as “4, very fresh”. The freshness classification performance of the SVM classifier and ANN models was compared in terms of the overall accuracy. Only 2% overall accuracy was different between the SVM classifier and ANN model at all level classifications (Fig 4). Thus, the SVM classifier accuracy was almost equal to that of the ANN model.

**Fig 5:**
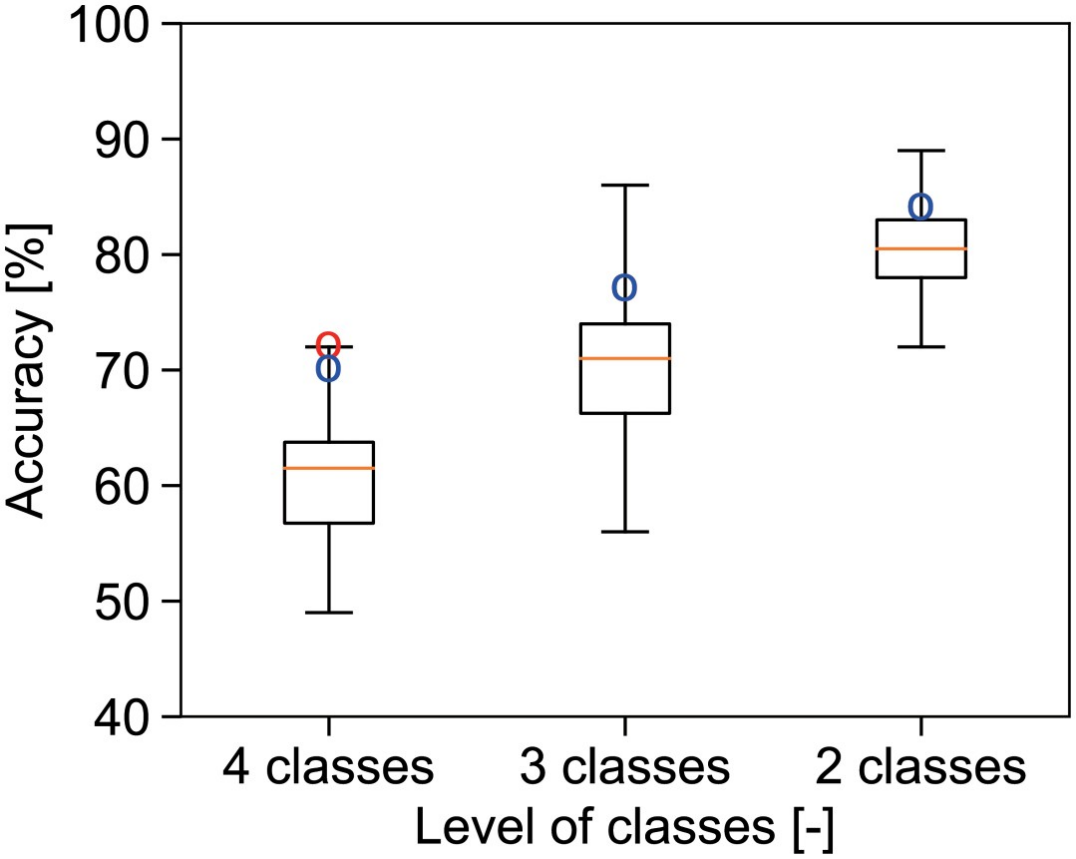
Accuracy sensory evaluation for test set by a panel and machine learning model. The true label is the rounded value of the average from all panel evaluations. Boxplot is the results of 12 non-trained panel, where whiskers indicate the minimum and maximum of the accuracy. Red and blue points: accuracy by SVM and ANN, respectively.

The individual evaluation results are shown in Fig 5, where the boxplot indicates the accuracy of individual panels. The true label is the rounded value of the average from all panel evaluations. The red and blue plots show the overall accuracy of SVM and ANN model prediction in Fig 4. The lower the class number, the higher the accuracy in both the machine learning model and panel evaluation. All the overall accuracy by SVM and ANN was within the range of maximum and 75% quantile in the boxplot. Thus, the overall accuracy of SVM and ANN was similar to that of individual evaluation. The performance of both the SVM classifier and ANN model were similar to the individual evaluations in terms of the accuracy of freshness evaluation.

## Discussion

Machine vision systems have been previously applied to the field of vegetable and fruit classification [20,27], using texture and color features to identify and describe damaged areas on fruits [50]. Moreover, color co-occurrence has been used to classify diseased and normal leaves [51] and citrus fruit [52]. In a previous study, local damage to strawberries was detected using local key point detectors, such as SIFT and ORB [39]. In another study, the freshness of fresh-cut iceberg lettuce was classified via convolutional neural networks [29]. Various image processing techniques have been used for feature extraction. In our study, we focused on the color and local features for ORB key point detection. The sensory evaluation score showed a positive correlation for a minimum value of gray, g, v, and L* and a negative correlation for six clusters in the ORB local features. Despite numerous combinations of image processing, our model showed that the possibility of color and ORB key point features would be useful for classifying the level of freshness of spinach leaves.

Here, we look at the detail of classification results. Fresh and very fresh were not separated in high accuracy (Fig 4(c) and 4(f)). One of the reason may come from lack of number of data. The 48 images of “Very fresh” may be too small compared to the 452 images of “Fresh”. In contrast, in three-class and binary classification, the classification results showed high overall accuracy of prediction over 77%. Thus, our model showed that the developed model enabled to class spinach freshness. In the present study, the smaller number of classes were, the higher accuracy were shown in both panel evaluation score and machine learning model. (Fig 5). One reason is explained by probability theory. If one randomly select one class in two classes, accuracy would be around 50% in binary classification. In the same random sampling methods, the accuracy would be around 33% in three-class classification and around 25% in four-class classification. Thus, the smaller number of classes are, the higher accuracy is easily shown. As Fig 5, the classification results were agree with the trend.

Fruit and vegetable freshness has been investigated from many points of view, including the chemical, physiological, and optical properties. Some studies focusing on the chemical and physiological conditions of vegetables and fruits have used destructive methods [7,8], which are unsuitable for quality prediction. Meanwhile, other studies have used near-infrared spectra and hyperspectral imaging. These technic enables to get spectra of non-visible wavelength (900–1700 nm). Non visible spectra information may not directly reflect the sensory evaluation of spinach freshness, since the sensory evaluation of freshness is based on subjective visual perception [53]. However, in our proposed model, images were used as inputs for both human sensory evaluation and machine learning. Wavelength in digital image is link to human visual wavelength (400–700 nm). Because the input was limited to visual images, prediction by machine learning model was close to subjective visual human evaluation. Evaluating freshness using images is a reasonable methodology to predict freshness based on visual perception. Thus, our modeling method is superior to that of near-infrared spectroscopy and hyperspectral cameras in terms of mobility and cost. Our proposed model setup was simple, easy to use, and required only smartphone images.

Some studies have attempted to predict sensory evaluation for food using labels with fixed true values judged by trained panels. For example, the rate of red color in fish skin is defined as the freshness index [19]. Cheese quality has been predicted using texture perceived by trained judges based on instrumental texture measurements [54]. Spinach freshness grade is predicted by learning the judgment of trained sensory panels [28]. Compared to these studies, our model focused on a vague target for calculating freshness in individual non-trained panels. Nevertheless, our model predicted the mean freshness value with overall accuracy of over 70% (Fig 4), which was similar to the accuracy of the panel evaluation (Fig 5). In the case of the 3- and 2-level classification, the prediction accuracy was 77% and 84%, respectively. These results indicate that freshness can be predicted using a machine learning model.

Although sensory evaluation is important for determining freshness, it is expensive, time consuming, and must be performed at a coordinated time in a single place. By contrast, the machine learning and image processing method can evaluate spinach freshness at any time from any location while remaining cost effective. Therefore, our model could be considered as a case study to demonstrate the applicability of food index prediction using smartphone images. The evaluation of freshness using a smartphone image would be useful for verifying the level of freshness of spinach in supermarkets, since a uniform freshness evaluation cannot be performed by sensory evaluation in different places at any time.

Our model is limited by the target of the non-trained panels. The panelists used in the presented study had the same age ranges (21–28 years; mean age, 23.3 years) and were from the same country. As such, the results of the freshness evaluation could change depending on the age and country of origin of the panelists, and other factors [4]. Thus, the results of sensory evaluation could change depending on the target consumer. Another limitation is a dataset of spinach. Our model is restricted to our dataset. Spinach leaf collected from different region and at different season and for different varieties need to be investigated to make a robust model. Multiple targets of consumers will need to be investigated in the future to develop a comprehensive consumer-based freshness evaluation model.

## Conclusions

This study developed classification models for spinach leaf freshness that predicted the mean value of sensory evaluation among panels. The machine learning model included color and local features to classify spinach leaf freshness. ORB was effective tools for extending feature of complicated local texture on spinach leaves. The prediction accuracy of this method was over 70%, which is similar to the accuracy of individual panel evaluation. These results suggest that images and machine learning have the potential to replace freshness evaluation by consumers’ targets. The proposed model can nondestructively and rapidly evaluate spinach freshness and be used from food supply thorough supermarkets to consumers to continuously check spinach freshness. Spinach leaves collected from wide variety of region would be investigated to make a robust model in the future.

## Supporting information

S1 File Code, dataset, and images are available at https://github.com/kento-koyama/Predicting-sensory-evaluation-of-spinach-freshness-using-machine-learning-model-and-digital-images. (DOCX)
