# Supplementary material for: Predicting sensory evaluation of spinach freshness using machine learning model and digital images
Source: PLoS One. 2021 Mar 19;16(3):e0248769. doi: 10.1371/journal.pone.0248769 (PMC7978266; doi:10.1371/journal.pone.0248769)
Supplement: S1 File — (DOCX) [file pone.0248769.s001.docx]

**S1 File**. Code, dataset, and images are available at https://github.com/kento-koyama/Predicting-sensory-evaluation-of-spinach-freshness-using-machine-learning-model-and-digital-images.
